# Undocumented Patients in the Emergency Department: Challenges and Opportunities

**DOI:** 10.5811/westjem.2019.7.41489

**Published:** 2019-08-12

**Authors:** Shamsher Samra, Breena R. Taira, Erika Pinheiro, Rebecca Trotzky-Sirr, Todd Schneberk

**Affiliations:** * Harbor-UCLA Medical Center, Department of Emergency Medicine, Torrance, California; † Olive View-UCLA Medical Center, Department of Emergency Medicine, Sylmar, California; ‡ Al Otro Lado Inc., Los Angeles, California; § LAC-USC Medical Center, Department of Emergency Medicine, Los Angeles, California

## Abstract

In the United States, undocumented residents face unique barriers to healthcare access that render them disproportionately dependent on the emergency department (ED) for care. Consequently, ED providers are integral to the health of this vulnerable population. Yet special considerations, both clinical and social, generally fall outside the purview of the emergency medicine curriculum. This paper serves as a primer on caring for undocumented patients in the ED, includes a conceptual framework for immigration as a social determinant of health, reviews unique clinical considerations, and finally suggests a blueprint for immigration-informed emergency care.

## INTRODUCTION

***Ms. G.S. is a 35-year-old woman presenting with “migraines.” She sits, tearful, clutching her head with both hands as she hunches over the foot of her bed. As the providers enter the room, her friend whispers, “She’s been under a lot of stress.” The patient explains that she is a single mother of three children working as a housekeeper. Work has been hard to find this month, and her client refused to pay her after she worked all day. After asking why she didn’t go to the police, she reveals that she is undocumented. Without the payment she was supposed to receive today, she cannot pay her rent and faces eviction. Her head has been hurting for weeks, but she has no health insurance and does not have a primary care provider. Her friend convinced her to come to the emergency department (ED) but she doesn’t want to incur another bill, so she would prefer to leave***.

The case of Ms. G.S. illustrates some of the many barriers that our undocumented patients face in achieving medical and social well-being. More than 11.3 million undocumented people currently reside throughout the United States.^1^ Among this population, 47% are women and approximately 9% are minors. The majority of undocumented individuals are from Mexico (56%), followed by Central America (15%), and Asia (14%).^1^ Nationally, the Hispanic undocumented population comprises 4% of the entire population but 4.8% of the workforce.^2^ Undocumented individuals have high rates of structural vulnerability compared to documented immigrants and are more likely to live below the federal poverty level (56% vs 32%). Also, compared to documented immigrants, more undocumented immigrants have not completed high school (52% vs 43%), and have poor English literacy (75% vs 53%). Almost 7% of all U.S. K-12 students have at least one parent who is undocumented, and one third of children of undocumented parents live in poverty.^3^

The aim of the Patient Protection and Affordable Care Act (PPACA), signed into law March 23, 2010, was to decrease the number of uninsured Americans through the expansion of public and private health insurance. The bill explicitly excluded undocumented residents in the U.S.^4^ Prior to the passage of the PPACA, it was estimated that undocumented residents made up 20% of the 46 million uninsured Americans.^5^ With the passage of the PPACA, few undocumented individuals gained eligibility for direct enrollment into health plans, although coverage improved slightly through funding of Federally Qualified Health Centers and employer-based coverage. Consequently, more than 45% of non-elderly undocumented immigrants are uninsured. As of 2017, non-citizen U.S. residents, including undocumented individuals and legal permanent residents, make up 7% of the U.S. population but approximately one quarter of the U.S. uninsured population. When healthcare is accessed, undocumented individuals report lower quality of health services including fewer doctor visits, lower rates of preventative testing, and lower perceived quality of care relative to U.S.-born Latinxs.^6^ Inequities in care may be compounded by language barriers in the setting of inadequate access to and utilization of interpreter services.^7^ Current federal immigration regulations risk exacerbating non-citizen reliance on ED care through increased barriers to alternative venues.^8^

Barriers to routine care increase dependence on public institutions and EDs. In California, 39% of undocumented individuals lack a usual source of healthcare other than the ED. They are also less likely to have had an ED visit over the preceding year compared to naturalized and U.S.-born citizens.^3^,^9^,^10^ Cost is often a point of contention in debates over healthcare for the undocumented.^11^ While robust and up-to-date statistics regarding healthcare utilization by undocumented individuals is limited, existing data strongly suggests that undocumented residents have lower per-capita healthcare expenditure than U.S. citizens. Furthermore, the undocumented population contributes to, but is ineligible to access, the Medicare Trust Fund, resulting in a large surplus of funds to this public healthcare-funding mechanism.^12^

Therefore, despite lower rates of healthcare utilization and expenditures compared to U.S. citizens, undocumented U.S. residents remain uniquely dependent on the ED for care. It is essential for emergency medicine (EM) providers to understand the unique health challenges and barriers to healthcare access faced by this population.

### Immigration as a Social Determinant of Health

**“**The social determinants of health are the conditions in which people are born, grow, live, work and age. These circumstances are shaped by the distribution of money, power and resources at global, national and local levels.”^13^ Immigration and undocumented legal status are important but often overlooked social determinants of health. Like other social determinants of health, immigration status both directly and indirectly impacts health and healthcare access. Some examples of the direct health effects of one’s legal status include unsafe work and living conditions, fear of detention and deportation, migration-related trauma, and barriers to accessing health-care. Undocumented status indirectly impacts health by limiting access to public service benefits, housing, preventative health screening, and other health promoting services.^14^ Yet among other social determinants of health, undocumented status is unique in that it is overtly criminalized and persecuted. Heightened crim-inalization of those without legal status, and their families, serves to compound the aforementioned barriers.

Further, the effects of anti-immigrant legislation and a culture of fear and distrust on healthcare utilization are well documented.^15^ Total well visits decrease, while acuity increases across a spectrum of contexts including psychiatric visits in California, pediatric ED visits in Georgia, or prenatal and well-child visits in Arizona.^16^–^18^ One in eight undocumented Latinx immigrants fears discovery and deportation when using the ED, which explains some of the sentiments fueling the pattern.^19^

#### Clinical Considerations in Caring for Undocumented Patients in the Emergency Department

There are several unique considerations to caring for undocumented patients in the ED. The following six cases provide examples and analysis of some of these unique situations.

##### Case 1

***Mr. E.B. is a 48-year-old man with a history of end stage renal disease on dialysis. He has suffered two cardiac arrests secondary to delayed dialysis and relies solely on the ED for his dialysis sessions. He is asymptomatic today but is afraid he will not be able to secure transportation to return to the ED before suffering another complication from delayed dialysis. He presents to the ED hoping to talk to social work about transportation for tomorrow’s dialysis session***.

Because they are ineligible for Medicare and Medicaid, undocumented residents are ineligible for routine dialysis. These individuals often rely on emergency dialysis in EDs funded through states’ emergency Medicaid funds. Dependence on emergency care services for a life-sustaining therapy creates significant medical and psychological distress for patients and their families and is associated with increased mortality.^20^,^21^ Compared to scheduled outpatient provision of this life-sustaining therapy, ED dialysis is 3.5 times as costly. Furthermore, lack of access to routine dialysis increases utilization of scarce emergency medical resources and results in lost work productivity for patients.^22^ Based on the negative medical, psychological and financial implications of existing practices, providers should advocate locally and nationally to ensure routine scheduled dialysis for all patients regardless of citizenship status.

##### Case 2

***Ms. J.G. is an 18-year-old female brought in by family for an acute acetaminophen ingestion secondary to suicidal ideation. She is in fulminant hepatic failure. She needs transfer to a transplant center to be evaluated for emergent liver transplant. One by one, you contact the local transplant centers. Each time the transplant surgeon accepts, but the transfer center states the request is on hold until it can “look further into funding options.” You then learn that she is undocumented***.

Lack of documentation status can exclude a patient from receiving an organ transplant. Although the United Network for Organ Sharing (UNOS) does not exclude patients based on citizenship status specifically, lack of health insurance precludes undocumented immigrants from being listed for organ transplantation.^23^ Few patients without health insurance are ever listed. Given the barriers to health insurance faced by non-citizens, undocumented patients are effectively excluded from consideration for transplant.^24^ Despite this, 3% of all organ donors in the U.S. are undocumented and most of their organs are transplanted into U.S. citizens.^25^ In an ethical analysis of this point, Wightman et al. write, “Any system that uses the organs of individuals who would themselves not be considered eligible for a transplant because of inability to pay is clearly unjust.”^26^ Economic analysis has demonstrated that the “break-even” point after which kidney transplantation is cheaper than ongoing dialysis is only 1.5–2.7 years.^27^ In addition, undocumented patients with Medicaid have post-transplant outcomes equal to that of U.S. citizens on Medicaid, refuting the argument that patients with limited financial resources are not able to adequately maintain their health post-transplant.^28^ In 2015, Illinois became the first state to use state funding to cover kidney transplantation costs for undocumented residents.^28^

##### Case 3

***Mr. S.F. is an 18-year-old male presenting the ED after he was assaulted while walking home from work. This is the second time he has been assaulted and is concerned for his safety. When asked if he’d like to file a police report he declines, fearing discovery of his immigration status***.

Undocumented groups are at risk for violent injury and reinjury because they are less likely to engage with law enforcement. Fear may prevent victims of violence from reporting crime or seeking other forms of support. Fear of deportation may be exploited to perpetuate both domestic violence and violence in the workplace in the form of human trafficking and/or unsafe or illegal work conditions. Recognizing this vulnerability, the federal government created the U-Visa as a part of the “Victims of Trafficking and Violence Protection Act of 2000,”^29^ which offers a path to legal residency to those who are willing to support law enforcement in the prosecution of the crime.

In cases where a patient’s citizenship status hinders communication and care, the emergency provider may consider articulating the confidentiality of the patient-doctor encounter, offering an introductory explanation of a U-Visa, and referral to either social work or appropriate local legal aid agencies. This intervention alone may reduce the likelihood of future victimization and offer the victim channels of support in an otherwise alienating environment.

##### Case 4

***Mr. J.S. is 39-year-old man who presents after a syncopal episode while working in the fields in the heat. He had been feeling lightheaded all day. After receiving intravenous fluids, he improves. His employer remains at bedside throughout her medical care, repeatedly interjecting into the conversation, and is reluctant to leave the room. When asked more pointedly, the employer steps out of the room and the patient divulges that his worksite does not allow water breaks. When asked if he wants to report this to local authorities, he states he was brought to the U.S. to work and is afraid of being fired and deported***.

Like Mr. J.S., 12 million people live in conditions of coerced labor or sexual servitude generating over 150 billion dollars in profit.^30^ Between 600,000–800,000 people are trafficked across borders globally, almost half under the age of 18 and the majority female. Between 14,000–50,000 people are trafficked into the U.S. every year. Undocumented migrants are disproportionately represented in the trafficked population and have higher barriers to safety than U.S.-born victims, including poor social support and fear of deportation. Many labor-trafficking victims (67%) and a large percentage of sex-trafficking victims (13%) are believed to be undocumented.^30^ Considering the barriers to healthcare for undocumented individuals, the ED visit represents an opportunity to identify and assist undocumented victims of trafficking.^31^,^32^

The identification of trafficking victims is obstructed by a multitude of factors including distrust of authority, fear of retaliation, and fear of deportation.^33^ When concerned, providers should seek to interview patients independently, build trust, and offer resources that may be used later if and when the victim feels comfortable seeking assistance. Specifically, providers may inform possible victims of the T-Visa program. Like the U-Visa, the T-visa offers temporary and possibly permanent visas to victims of trafficking who agree to assist law enforcement in the identification of traffickers.

##### Case 5

***Mr. J.F. is a 35-year-old man presenting with recurrent headaches since immigrating to the U.S. from El Salvador. He and his brother owned a tire repair business, which he left behind after his brother was killed because they couldn’t comply with increasing extortion demands from a local gang. He endorses frequent panic attacks and flashbacks, and fears deportation back to his community where his family members continue to receive threats***.

Emergency providers may encounter individuals or families who are undocumented and facing or fleeing torture in their home country. Many recent migrants from Central America and Mexico cite the burden of gang violence, political violence, and torture as reasons for fleeing their home countries. A survey of migrants by Médecins Sans Frontiers found that over half of migrants reported violence as the primary driver of emigration while 68.3% endorsed being victims of violence en route.^34^ Past exposure to political violence may manifest clinically as depression, post-traumatic stress disorder, panic disorders, chronic pain, and impaired physical functioning.^29^,^34^ Despite the high levels of torture and violence in Mexico and Central America, few submit asylum applications.^35^ ED providers should consider the burden of torture and political violence in clinical encounters and consider referral to trusted legal aid groups to seek asylum, programs for torture victims, and psychosocial support services.

##### Case 6

***Ms. R.Z. is a 22-year-old woman brought in by U.S. Border Patrol officers who found her in the desert after she crossed from Mexico. She is hypothermic on initial examination. Her pregnancy test is positive. On further discussion she endorses sexual violence during her migration, inflicted by the smuggler she had paid to guide her from Guatemala***.

While the total number of individuals successfully crossing the U.S. border is unknown, the number of apprehensions at the U.S. border was approximately 415,000 in 2016.^36^ The vast majority occur at the southern border and are of individuals from Mexico or Central America. The factors that underlie the ebb and flow of migration rates are complex, including international disparities in wealth, violence, and border militarization. Increased expenditures on border militarization have led to more perilous journeys for migrants.^37^ Prior to 1994, border-crossing deaths were a rare occurrence. Progressive militarization of the border pushes migrants to pursue more treacherous routes, exposing them to extremes of hot and cold.^38^ Common causes of death and morbidity include drowning, dehydration, motor vehicle accidents, and violence from law enforcement.

According to U.S. federal statistics, 307 immigrants died during border crossings in 2014 alone and over 6,000 have died since 1998. The Mexican government, however, estimates nearly triple that of the U.S. government.^37^ The health risks of migration begin long before the hazardous U.S. border crossing. Despite being guaranteed “a right to receive healthcare provided by either the public or private sector, regardless of their migratory status” by the Mexican Migrant Law, most migrants from Central America travel long distances across Mexico without access to medical care. Interviews with migrant women at Mexico’s southern border found that 28% had transactional sex, 8.3% had been sexually assaulted, and 9.2% had suffered sexual harassment.^39^ As many as six in ten women may be sexually assaulted at some time during their trip.^40^

### Creating an Immigration-Informed Emergency Department

“…[T]he ED is singled out as the only component of the medical system and, in this case, *the only component of the entire social welfare system*, that is protected by law for many of the most disadvantaged.”^41^

In the face of daunting political, social, and economic forces that threaten the health of undocumented populations, there are tangible steps ED providers may take to promote health equity. Here we offer a blueprint for creating an immigration-informed ED (Figure). Improving the care and health of undocumented populations begins by training providers that immigration status is a modifiable social determinant of health. Trainees in EM along with other hospital staff should be encouraged to reframe discussions on immigration status from a polarizing political topic to one that directly impacts patient care. Understanding the demographic composition of one’s ED, hospital, and local community is vital. This provides a basis for understanding cultural and structural conditions that shape ED visits including perceptions of the healthcare system, traditional practices, and factors driving emigration from countries of origin.

Clear communication between patients and providers is critical to compassionate and equitable care for undocumented patient populations. To improve the quality, safety, and satisfaction of patient care – including developing trust and promoting treatment adherence – it is important to identify language barriers and use professional interpreters. This is not merely a formula for improved care but is also legally enshrined in Title VI of the 1964 Civil Rights Act of the Department of Health and Human Services, which states: “No person may be subjected to discrimination on the basis of national origin in health and human services programs because they have a primary language other than English.”^42^ Undocumented patients may be denied access to health promoting services and treatment modalities that are accessible to other patients, including public programs, nursing care, and particular medical therapies following discharge from the ED.^43^ ED providers should work with social workers and community-based providers to provide tailored care plans.

Immigration relief is the adjustment of immigration status to a legal category that allows the person to stay in the U.S. without fear of deportation. Adjustment of legal status has multiple benefits that contribute to long-term improvement in health including stabilization of socioeconomic status and eligibility for health insurance (Table 1). Providers serving undocumented patients should be aware of the types of immigration relief most relevant to ED patient care (Table 2). Although the intricacies of immigration relief eligibility are beyond the purview of ED providers, understanding these basic categories will prompt particular attention to immigration status in specific patient populations and disease presentations. Providers should be aware that a multitude of barriers have been erected to limit asylum and immigration relief and take care not to promise immigration-status adjustment. Instead, cases should be referred to qualified legal advocates to avoid misinformation. Formation of medical-legal partnerships, as discussed below, can improve identification of eligible cases.

Strengthening ties with local, community-based organizations and medical legal partnerships can facilitate linking undocumented patients to social services. Community-based organizations understand challenges faced by undocumented groups, have organized resource databases, and may be able to facilitate successful completion of the intended discharge plan. Coordination with local legal aid groups may streamline referral for victims of trafficking, violence, and others that may qualify for legal status change or benefit from legal rights education. Vetting of community partners is imperative because of the high prevalence of predatory legal service providers.^44^

Given the vulnerability of this patient population, and impoverished communities more generally, safety-net facilities may consider building medical-legal partnerships to integrate legal services into the clinical space.^45^ In addition to legal status adjustment, immigration legal providers may alleviate fear and anxiety by educating patients about their legal rights in the home, workplace, and public. The feasibility of these interventions may be limited by time constraints of ED providers. Providers may consider partnerships with social workers, community-based organizations, and local student volunteers to establish more robust systems of social screening, referral and case management out of the ED to facilitate referrals.^45^

By bearing witness to the human impact of anti-immigration legislation on patient health, ED providers make excellent advocates on the local and national level. Political policies enforcing the detention, criminalization and deportation of undocumented populations cause increased fear of entering the public sphere and fear of engaging with social services, including healthcare.^19^,^46^ Consequently, undocumented populations are at increased risk of foregoing preventative and potentially life-saving medical care due to fear of detention. ED providers should advocate for policies that ensure sanctuary spaces for all patients, including those who are undocumented.^47^ Central to this designation are hospital and health-system policies that support hospital staff in limiting cooperation with immigration agencies and agents. Sanctuary city and state policies such as those passed in California may offer guidance in policy creation, advocacy and implementation.^48^ Beyond formal policies, hospitals and EDs should communicate their acceptance and support of undocumented communities through hospital signage, local community outreach, and partnership with local, community-based organization and trusted civil society groups.

Existing political policies that limit access to health-promoting services and promote detention or criminalization are deleterious to the health of the undocumented patient population. For healthcare providers advocacy against the “illegality of humans” is inextricably intertwined with professional ethics and the principle of health as a fundamental human right. Emergency physicians, healthcare providers, and health systems striving for health equity should engage in local and national organizing to address these barriers to care, by advocating for paths toward legal status and funding to ensure equitable care such as routine dialysis and organ transplantation.

Offering a patient-centered lens to local organizing efforts, creating local immigration-focused provider groups, promoting the creation of sanctuary spaces, developing immigration-informed EDs, and formulating patient-centered position statements among professional organizations are just some of the avenues through which physicians may engage in advocacy to address immigration-related barriers to health. Inclusion of immigrant and undocumented communities in these organizing efforts is essential to ensure that advocacy efforts align with needs.

## CONCLUSION

“*The physicians are the natural attorneys of the poor, and the social problems should largely be solved by them.”*

Rudolf Virchow

The subject of citizenship and legality of populations is complex, contentious, and dynamic. It is clear that lack of citizenship negatively impacts access to healthcare and health. Similarly, the idea of “illegality of persons” contradicts the professional and societal obligations of healthcare providers. The cases highlighted here illustrate how existing systems fail to meet the needs of undocumented patients. We hope this primer informs clinicians in the ED about the multiple levels of barriers undocumented patients face and suggests potential provider- and system-level changes to counteract exclusionary policies and promote health equity. As is often the case, it falls upon healthcare providers to transcend existing norms to secure the medical and structural conditions requisite to the health of our patients.

## Figures and Tables

**Figure 1:**
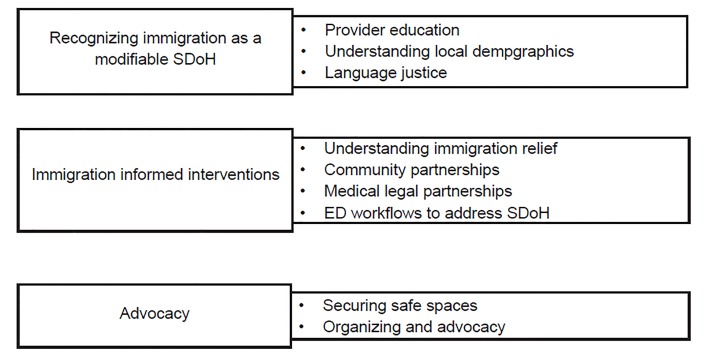
Components of an immigration-informed emergency department. *SDoH*, social determinant of health; *ED*, emergency department.

**Table 1:** Benefits of obtaining legal immigrant status (immigration relief).

Increaseding access to safe, legal employment, with increased opportunities for enrollment in employment-based health insurance. Improved socio-economic status with resultant stabilization of life situation. Possible eligibility for enrollment into federal or state programs for education or health services, i.e., Medicaid. Self-sufficiency and independence from exploitative living and work environments.

**Table 2:** Clinically relevant immigration-relief scenarios.

Immigration relief	Clinical scenario	Suggested phrasing
U-visa	Undocumented victims of certain serious crimes	I want to tell you that certain victims of serious crimes in the U.S. may qualify for immigration relief.
T-Visa	Survivors of human and labor trafficking	Some patients in your situation may be eligible for immigration relief and services.
Special Immigrant Juvenile Status (SIJS)	Undocumented patients under the age of 21 who have been abandoned, abused or neglected by one or both parents, including children in foster care, in guardianship proceedings, or on probation. Also includes those under 18 living with a single parent.	Some patients in your situation may be eligible for immigration relief. I wanted to tell you that if you qualify for immigration relief, it is important to talk to an immigration attorney as soon as possible.
Asylum and Convention against torture (CAT)	Undocumented patients who experienced persecution or torture in country of origin	Some patients who have suffered serious harm or threats of harm in their home country may be eligible for immigration relief.
